# Implantoplasty Improves Clinical Parameters over a 2-Year Follow-Up: A Case Series

**DOI:** 10.3390/medicina58010113

**Published:** 2022-01-12

**Authors:** Orlando Martins, Philipp Sahrmann, João Ramos, Francisco Caramelo, Sérgio Matos, Isabel Poiares Baptista

**Affiliations:** 1 Dentistry Department, Institute of Periodontology, Faculty of Medicine, University of Coimbra, 3000-075 Coimbra, Portugal; sergiomatos1@sapo.pt (S.M.); isabelpoiaresbaptista@gmail.com (I.P.B.); 2 Dentistry Department, Institute of Oral Medicine and Surgery, Faculty of Medicine, University of Coimbra, 3000-075 Coimbra, Portugal; 3 Clinic for Periodontology, Endodontology and Cariology, University Center of Dental Medicine Basel, 4058 Basel, Switzerland; philipp.sahrmann@unibas.ch; 4 Dentistry Department, Institute of Operative Dentistry, Faculty of Medicine, University of Coimbra, 3000-075 Coimbra, Portugal; joaoctramos@sapo.pt; 5 Laboratory of Biostatistics and Medical Informatics IBILI, Faculty of Medicine, University of Coimbra, 3000-548 Coimbra, Portugal; fcaramelo@fmed.uc.pt

**Keywords:** peri-implantitis, implantoplasty, case reports, dental implants

## Abstract

*Background and Objectives*: Peri-implantitis treatment is still undefined. Regenerative treatment is expensive and technically demanding due to the need to handle biomaterials, membranes and different methodologies of decontamination. Resective treatment and implantoplasty might be a viable solution. This case series presents a 24 month retrospective observational study of 10 peri-implantitis patients treated with implantoplasty. *Materials and Methods*: In the present case series, 10 peri-implantitis patients (20 implants) were treated with a resective approach and implantoplasty. Previous to implantoplasty, all patients underwent non-surgical treatment. This surgery consisted in a full-thickness flap and implant surface exposure. The exposed non-osseointegrated implant body was submitted to implantoplasty. The flap was apically repositioned and sutured. Patients were accompanied for 24 months. *Results*: The mean initial probing depth (PD) (PD = 5.37 ± 0.86 mm), bleeding on probing (BoP = 0.12 ± 0.06%) and suppuration (Sup = 0.01 ± 0.01%) decreased significantly at the 12 month evaluation (PD = 2.90 ± 0.39 mm; BoP = 0.01 ± 0.01% and Sup = 0.00 ± 0.00%). Between the 12 and 24 month evaluations, there were no significant clinical changes (PD = 2.85 ± 0.45 mm; BoP = 0.01 ± 0.01% and Sup = 0.00 ± 0.00%). Mucosal recession (MR) had a significant increase between the baseline and the first 12 months (0.69 ± 0.99 mm vs. 1.96 ± 1.33 mm), but there were no significant changes between the 12th and 24th month (1.94 ± 1.48 mm). The success rate was 100% without implant fracture or loss. *Conclusions*: Resective surgery and implantoplasty might be a valid option in some specific peri-implantitis cases. Properly designed clinical trials are needed to confirm this possibility.

## 1. Introduction

Peri-implantitis (PI) is defined as a biofilm-induced progressive bone loss due to inflammation of the peri-implant tissues. In the absence of initial data, clinical symptoms of peri-implantitis are the presence of bleeding on probing (BoP) and/or suppuration (Sup), probing depth (PD) ≥ 6 mm and bone levels ≥ 3 mm apical of the most coronal portion of the intraosseous part of the implant [1]. A 2008 consensus report indicated a prevalence of PI in 28–56% of the patients and 12–40% of the implants [2] and a recent meta-analysis estimated a PI weighted mean prevalence of 22% [3]. This variability might be partially related to different case definitions confirmed by a recent study on an inter-rater agreement in the diagnosis of peri-implant diseases [4]. Guidelines for peri-implantitis treatment remain inconclusive. A recent review concludes that the available evidence does not allow any specific recommendation for the treatment of PI [5]. Long-term results for a PI regenerative approach have limited evidence [6], and there is no strong evidence to suggest a respective treatment modality with superior outcomes [7], while prognosis in terms of resolution is generally unsatisfactory. Regenerative approaches, while showing an enhanced morbidity, are more costly than non-regenerative approaches due to the additional use of biomaterials and membranes. The peri-implant defect morphology also influences the outcome of regenerative treatment of peri-implantitis. Circular self-containing class (Cl) Ie defects are promising for regeneration, unlike Cl Ib and Cl Ic, which are considered less favorable [8]. As an alternative to the regenerative approach, resective interventions constitute a cheaper and—regarding the requirements of the bone defect morphology—less demanding intervention.

Implantoplasty (IP), granulation tissue removal and peri-implant pocket reduction are the basic steps of resective peri-implantitis surgery. Implantoplasty comprises the removal of the windings and rough surface of the contaminated implant and a subsequent polishing. Additionally, bone recontouring might be needed in order to allow a post-surgical anatomy to make the access for oral hygiene measures easier. Finally, apical repositioning of the mucosa flap is recommended to expose the new-shaped surface to daily brushing [9,10,11]. Likewise, this approach is also indicated if the patient shows insufficiently controlled risk factors contradicting regenerative approaches. Some data point to the use of specific burs in order to obtain an implant roughness not attractive to plaque formation [12,13]. Presently, there is a very limited data regarding to the clinical outcome of implants treated with implantoplasty [11,14]. The aim of this case series is to contribute to a better understanding of the clinical outcome of resective surgery and implantoplastythrough a retrospective observational analysis.

## 2. Materials and Methods

### 2.1. Study Design

Ten partially and fully edentulous patients (20 implants), mean age 60.9 ± 11.0 years old, were diagnosed with peri-implantitis, treated and included in this retrospective case analysis. This study was conducted in accordance with the 1975 Declaration of Helsinki of 1975 as revised in 2013. The study protocol was approved by the Medical Faculty of Coimbra ethical committee (CE 031.2019). All peri-implantitis surgeries were performed by the same surgeon (OM) and consisted in a resective approach and implantoplasty made in two private clinics and one university hospital. Previous to IP, all patients with periodontal inflammation were submitted to periodontal treatment and underwent a customized periodontal maintenance therapy. Before surgical treatment, all patients underwent non-surgical therapy in order to reduce signs of inflammation on implant sites in a time period of 2–6 weeks before surgery. This non-surgical treatment consisted in an implant scaling with a titanium curette (Depeller^®^, Rolle, Switzerland) and glycine powder (EMS^®^, Nyon, Switzerland) and subgingival irrigation with povidone iodine 10% (Betadine^®^, Lisbon, Portugal). All patients were rehabilitated with more than one implant (Table 1).

Discomfort during mastication and profuse bleeding when brushing was mentioned by all patients, with the consequent restrain to perform oral hygiene. Oral malodour was also mentioned. For this retrospective case series, peri-implantitis was defined as PD ≥ 6 mm and/or radiographic bone loss ≥ 3 mm in conjugation with profuse bleeding [15]. All included patients respected these parameters. At the time of inclusion, each patient revealed at least one class II supracrestal defect associated with an intrabony two-wall defect class Id or only supracrestal component class II [16]. These anatomic characteristics were confirmed during surgery. The included patients had to fulfil the following inclusion criteria: (a) no implant mobility, (b) only class II supracrestal defect or associated with intrabony two wall defect class Id [16], (c) no occlusal overload, (d) good level of oral hygiene (plaque index < 1 [17]), (e) less than 10 cigarettes per day and (f) no systemic diseases that could influence the therapy outcome (diabetes (HbA1c < 7), osteoporosis, bisphosphonate medication or cancer). No patient was previously submitted to peri-implantitis surgery. All participants were given a detailed description of the procedure and all signed the informed consent form.

### 2.2. Clinical Evaluation

Peri-implant clinical evaluation was made using a periodontal probe (PUNC-15 Hu-friedy, IL, USA) evaluating six sites per implant (mesiobuccal, midbuccal, distobuccal, mesiopalatal, midpalatal and distopalatal). The following peri-implant measures were performed: (a) probing depth (PD) measured from the gingival margin till the bottom of the probable peri-implant pocket, (b) mucosal recession (MR) measured from the implant shoulder (IS) to the mucosa margin (MM). The measured distances were rounded off to the nearest millimeter. Bleeding on probing (BoP) and suppuration (Sup) were assessed within 15 s after probing [18]. Data were collected by a specialist in periodontology (OM) at baseline, 12 and 24 months post-operative.

### 2.3. Surgical Procedure

**Figure 1:**
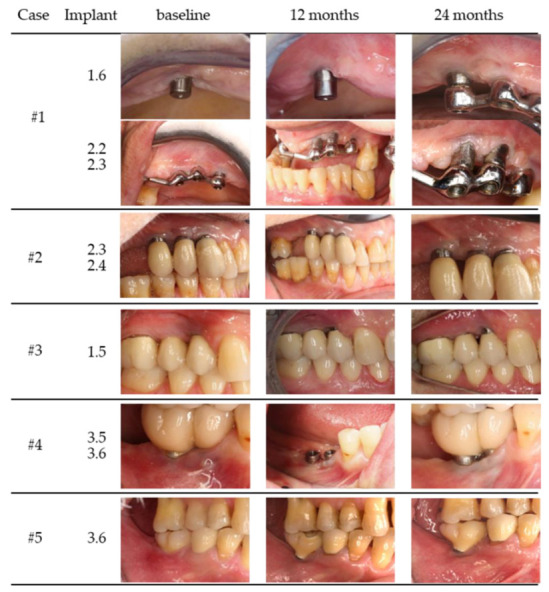
Clinical images of case 1–5 (baseline, 12 and 24 months).

After local anesthesia, intrasulcular incisions on the peri-implantitis-affected implant were performed and extended to one or two teeth on both sides to allow a good direct access to the peri-implant defect. Then, oral and vestibular full-thickness mucoperiosteal flaps were raised and the implant was exposed. Peri-implant granulation tissue was removed by using a Gracey curette (5/6 or 7/8 Gracey curette, Hu-Friedy, IL, USA). The surgical area was rinsed with saline solution. A peri-implant bone recontour was performed in order to remove vertical components using a rose-bur under abundant water cooling (Komet^®^, Brasseler GmbH, Lemgo, Germany), and the final bone contour was performed with bone chisels. The implant surface was polished (implantoplasty) using a sequence of round diamond rotatory burs at 200.000 rpm with the following sequence: blue (40 µm) and yellow (15 µm) (Coltène/Whaledent AG-Diatech, Altstätten, Switzerland). The final polishing was done with an Arkansas stone torpedo shaped aluminium oxide. All of the process was performed under copious irrigation with sterile saline solution. Finally, the implant surface and peri-implant tissues were irrigated with abundant saline solution. After internal mucotomy to thin out the soft tissues and a final spray with the glycine air flow system, the flaps were repositioned apically and sutured with modified mattress suture using a monofilament suture 5-0 (Seralon^®^, Serag-Wiessner, Germany) (Figure 1). Post-operative pain was controlled with ibuprofen 600 mg twice daily (5 days) and paracetamol 1000 mg tid (SOS). Patients were instructed to rinse with chlorhexidine 0.12% twice daily (Eluperio^®^, Pierre Fabre, France) until the sutures were removed after 10–15 days. During the first year after the implantoplasty, patients were recalled in an interval of 3 and 4 months. Afterwards, the recall frequency varied from 3 and 6 months. Patients were placed on an individually tailored maintenance periodontal program according to the patient’s risk profile [19]. During this period, the oral hygiene was assessed by the modified peri-implant plaque index (mPI) [20]. Oral hygiene reinforcement and tooth and implant cleaning were performed at each visit using titanium curettes, rubber cups and gauze with polishing paste.

These cases were followed for 24 to 64 months after the implantoplasty procedure. This case series included 10 patients with a total of 20 implants, 9 maxillary and 11 mandibular, with 4 to 9 years in function prior to peri-implantitis diagnosis, (mean of 6.95 ± 1.50 years). All patients were rehabilitated with more than one implant, but only two patients had their total number of implants treated with implantoplasty. Accordingly, a total of 11 mandibular and 9 maxillary implants were treated. The implants were restored with both screwed crowns and bar supported overdentures.

### 2.4. Statistical Analysis

Statistical analysis was performed by an independent statistician using a commercially available software (IBM^®^ SPPS^®^ v24). The patient was used as a statistical unit. The mean and standard deviation were calculated for all parameters. Normal distribution was tested with Shapiro–Wilk test. Statistical changes over time were tested with the Wilcoxon test. The level of significance was 0.05. The primary outcome was the final PD. Secondary outcomes were BoP, MR and Sup.

## 3. Results

**Figure 2:**
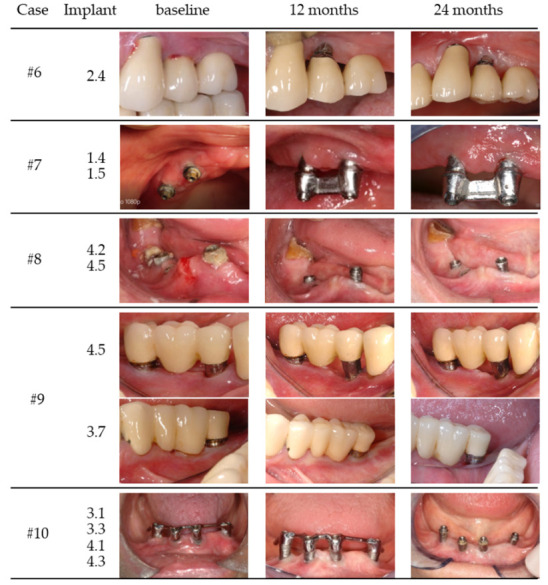
Clinical images of case 6–10 (baseline, 12 and 24 months).

In all patients, the wound healing was uneventful (no allergic reactions, abscesses or infections) (Figure 1 and Figure 2).

During the maintenance program, patient compliance was confirmed by the absence of visible plaque surrounding the implants (mPI ≤ 1) [20]. During the 24 months evaluation no implant fractured. No implant was lost or surgically retreated during the entire follow-up period. All 20 implants included in this retrospective analysis had an initial PD ≥ 6 mm on at least one peri-implant site before non-surgical pre-treatment. Before surgery, the mean PD of the treated implants was 5.37 ± 0.86 mm. One year after surgery, the PD values decreased significantly to 2.90 ± 0.39 mm. Compared to baseline, the 12 month evaluation also revealed a significant decrease for BoP (0.12 ± 0.06% vs. 0.01 ± 0.01%) (*p* = 0.005), Sup (0.01 ± 0.01% vs. 0.00 ± 0.00%) (*p* = 0.027) and an MR increase (0.69 ± 0.99 mm vs. 1.96 ± 1.33 mm) (*p* = 0.005).

At the 24 month evaluation, all clinical parameters had no statistically significant changes compared to 12 months. However, when comparing the baseline and the 24 month evaluation PD, BoP and Sup had statistically significant lower values and MR significantly higher. At both 12 and 24 months, one patient revealed a PD = 5 mm without bleeding-on-probing both one and two years after intervention. All the remaining patients showed PD values ≤ 4 mm. The clinical findings are summarized in detail in Table 2. No implants were lost during the 24 month evaluation, although some implants lost more than 50% of supporting bone (Figure S1—Supplementary Material).

## 4. Discussion

This case series assessed the clinical effect of resective surgery and IP over 24 months. The clinical parameters PD and BoP significantly improved 12 months after the intervention, in comparison to the baseline. This finding was sustained by the data of the 24 months follow-up period. There was an increase in MR, mirrored in an implant surface exposure. The significant improvement of PD and BoP at 12 months corroborated the results published by Romeo et al. (2005) despite these authors having used a different bleeding index, the modified sulcus bleeding index according to Mombelli et al. (1987), which resulted in a decrease from 2.83 ± 0.47% to 0.37 ± 0.58%. PD reduction is primarily attributable to the mucotomy procedure and apical repositioning of the flap. In a 12 month evaluation, our PD results (2.90 ± 0.39 mm) were slightly lower than those of Romeo et al. (3.43 ± 0.94 mm). One possible explanation for this difference might be the meticulous thinning of the mucosa in this case series. Probing around implants might also be influenced by the profile of the abutments and implants as well as the shape of the prosthetic component [21]. By employing this surgical technique, our implant survival rate was 100% and no mechanical problems such as deformation or broken implant were detected. Medium- to long-term results after implantoplasty are rare, and survival rates range from 100% at 24 months [22] to 97.9% or 100% at 36 months [11,14] and 87.2% at 108 months [14]. Our clinical results are in agreement with those data with a survival rate of 100%. In addition, we achieved the clinical resolution of peri-implantitis since no PD ≥ 6 mm were registered during the follow-up period. Pommer et al. (2016) referred the mean period between IP and implant loss after implantoplasty of 4.7 ± 1.6 years, with the first reported loss occurring 2 years after treatment. This case report includes no implant loss; however, these data should be interpreted with caution due to the fact that the number of cases presented is very limited.

The peri-implantitis definition used in this case report was the one reported by Renvert et al., 2018. One of the presented cases had a final PD = 5 mm (case #5) at one site of the treated implant without BOP, thereby indicating a healthy peri-implant situation [23]. The respective implant was placed next to the natural tooth with the bone crest in its regular position, more coronal than the treated implant. The position of the bone crest made it impossible to perform an apically repositioned flap without compromising the periodontal attachment of the natural tooth, a situation that non-seldomly compromises a perfect outcome in terms of osteotomy and perfect access to the most apical parts of the implant surface for smoothening with rotatory instruments [22].

With regard to an ongoing discussion about the stability of the implant after implantoplasty, data in the published literature are still rather scarce. The biomechanical stability of implants treated with IP might be affected since the implant strength is derived from the wall thickness [24]. While the hypothesis that removal of implant material from the shoulder area should result in an enhanced risk for fractures in loaded implants sounds logical, the literature is still inconclusive. Remarkably, several in vitro studies do not report any change in the implant’s stability after implantoplasty [12,25], while clinical studies assessing implantoplasty have not reported on respective fractures so far [26,27]. Despite IP producing a minimum diameter reduction (0.1–0.2 mm) [28], there are no conclusive results relating implant strength and different implant diameters (4.7, 4 and 3.75 mm) [12,29,30].

Regular maintenance and adequate accessibility to achieve plaque control are essential to avoid the relapse of peri-implant disease with the ultimate implant loss [31,32,33]. Our maintenance program and patient compliance were fundamental to achieve these results since we know that compliance and regular professional control are essential to obtain peri-implant soft tissue health [31]. A recent study identified full-arch rehabilitations as having 16.1 times more risk for PI than single rehabilitations [34], most likely due to the difficulty to perform an adequate oral hygiene. This might indicate that full rehabilitated patients should have adequate prosthesis design and probably more restrictive maintenance care program. Like Romeo et al. (2005), we included smokers and partial/total edentulous patients, both reflecting the daily clinical reality and the indication for this resective treatment modality. In the present study, two patients smoked five and nine cigarettes/day and showed a maximum cumulative smoke consumption of 164.55 packs/year. Smoking is an important factor that has been reported as having an influence on the treatment outcome [35,36]. However, in accordance with a recent study that included 6 smokers in a total of 25 patients, smoking had no effect on implantoplasty treatment outcomes [22]. In the present cases, there was no indication that smoking had any influence on the surgical outcome after IP since both smoking patients had peri-implantitis resolution. However, the small number of smokers does not allow any strong conclusion.

One of the most crucial steps of PI surgical regenerative approach is implant surface decontamination. Implantoplasty, however, is performed with the aim to completely remove the surface morphology of the osseodisintegrated implant surface. Thus, separate surface contamination is not a crucial issue. Despite IP being an available mechanical method to decontaminate the implant surface, other mechanical methods are also available, and a recent RCT showed no radiographic and clinical differences between IP and glycine air abrasion for the surgical treatment of peri-implantitis [37]. Final polishing of the instrumented surfaces reduces the implant roughness; therefore, the bacterial biofilm formation and maturation [38,39] and the risk of re-infection of the treated sites is hampered [40]. Mucosal recession might be a concern in the aesthetic zone. However, in posterior areas or in the case of overdentures, this is not a problem, and it might be well accepted by the patient if this fact is previously explained.

One of the—absolutely intended—consequences of implantoplasty is mucosal recession and exposure of the smoothened implant surface areas. The exposure of the implant surface allows a better plaque control. The accessibility for plaque control is extremely important since biofilms are considered the primary etiologic reason for peri-implantitis, while poor plaque control is considered a major risk factor for peri-implantitis [1,35].

The aesthetic problems related to titanium debris resulting from IP were also reported [40]. However, in this case series, no titanium pigmentation was observed in the mucosal margin. This might have happened due to the fact that internal mucotomy to thin out the soft tissues and a final spray with the air flow system were performed after IP. In order to improve clinical data related to IP, we consider properly performed studies important to evaluate the potential biotoxicity effects of debris generated during IP procedure and the possible interference with the clinical outcome.

## 5. Conclusions

Within the general limitations of a case series, our results suggest that resective peri-implant surgery with osteoplasty, implantoplasty and an apically positioned flap is a safe and possible way to treat peri-implantitis. Mucosal recession with the esthetic problems might limit applicability in the esthetic area.

## Figures and Tables

**Table 1:** Patients and implants data.

Case	Gender/Age	Smoking Status (Y/N)	Periodontal Disease (Y/N)	Health Status	Implant Position	Years in Function	Prosthesis/Fully (FE) or Partially (PE) Edentulous	Total Number of Implants (Position)
#1	female/62 year	N	Y	Healthy	1.6	6	bar overdenture/PE	6
					2.2	8		(1.3, 1.4, 1.6, 2.2, 2.3, 2.6)
					2.3			
#2	female/66 year	N	Y	Healthy	2.3	9	cemented single unit/PE	10
					2.4	9		(1.4, 1.5, 1.6, 2.3, 2.4, 2.5, 3.6, 3.7, 4.6, 4.7)
#3	male/47 year	Y	Y	Heathy	1.5	5	cemented single unit/PE	2
		(5 cig/d)						(1.4, 1.5)
#4	female/51 year	N	N	Healthy	3.5	6	screw type fixed partial/PE	2
					3.6	6		(3.5, 3.6)
#5	male/60 year	N	Y	Healthy	3.6	4	screw type single unit/PE	2
								(3.6, 4.6)
#6	male/65 year	N	Y	Healthy	2.4	7	Cemented bridge/PE	4 (1.4, 1.5, 2.3, 2.4)
#7	female/75 year	N	Y	Anti-HT	1.4	6	bar overdenture/FE	6
					1.5	6		(1.5, 1.4, 2.4, 2.5, 3.2, 4.2)
#8	male/43 year	N	Y	Healthy	4.2	5	hybrid overdenture/FE	13
					4.5	5		(1.1, 1.2, 1.4, 2.3, 2.4, 2.7, 2.8, 3.1, 3.4, 3.6, 4.2, 4.5, 4.6)
#9	female/60 year	Y	Y	Anti-coagulation	4.5	8	cemented bridge/PE	3
		(9 cig/d)			3.7	9	Single unit but bridge/PE	(3.7, 4.5, 4.7)
#10	female/80 year	N	Y	Healthy	3.3	8	bar overdenture/FE	
					3.1			4
					4.1			(3.3, 3.1, 4.3, 4.1)
					4.3			
Mean ± SD						6.95 ± 1.50		

Legend—Y: yes; N: no; cig/d: cigarettes/day; SD: standard deviation; Anti-HT: anti-hypertension.

**Table 2:** Clinical results and statistical analysis.

Parameters		Baseline	12 Months	24 Months
PD (mm)	$\bar{x}\;\pm s$	5.37 ± 0.86	2.9 ± 0.39	2.85 ± 0.45
	min/max	4.17/7.50	2.50/3.50	2.08/3.67
	IC95%	[4.75; 5.98]	[2.63; 3.18]	[2.53; 3.18]
	*p*	0.005		
	*p*		0.796	
	*p*		0.05	
	treatment effect	−2.52 IC95% [−3.17; −1.86]		
MR (mm)	$\bar{x}\;\pm s$	0.69 ± 0.99	1.96 ± 1.33	1.94 ± 1.48
	min/max	0/2.92	0.17/4.17	0.17/4.67
	IC95%	[−0.01; 1.4]	[1; 2.91]	[0.88; 3]
	*p*	0.005		
	*p*		0.673	
	*p*		0.005	
	treatment effect	1.25 IC95% [0.51; 1.98]		
BoP (%)	$\bar{x}\;\pm s$	0.12 ± 0.06	0.01 ± 0.01	0.01 ± 0.01
	min/max	0.01/0.17	0.00/0.04	0.00/0.03
	IC95%	[0.08; 0.17]	[0.00; 0.02]	[0.00; 0.02]
	*p*	0.005		
	*p*		0.344	
	*p*		0.005	
	treatment effect	−0.11 IC95% [−0.15; −0.07]		
Sup (%)	$\bar{x}\;\pm s$	0.01 ± 0.01	0.00 ± 0.00	0.00 ± 0.00
	min/max	0.00/0.03	0.00/0.00	0.00/0.00
	IC95%	[0.00; 0.02]	[0.00; 0.00]	[0.00; 0.00]
	*p*	0.027		
	*p*		1.000	
	*p*		0.027	
	treatment effect	−0.01 IC95% [−0.02; 0.00]		

Legend—PD: probing depth; MR: mucosal recession; BoP: bleeding on probing; Sup: suppuration; $\bar{x}$ : mean; s: standard deviation; IC: confidence interval; *p*: *p*-value; mm: millimeters; treatment effect: difference between baseline and 24 months results.

## Supplementary Materials

The following are available online at https://www.mdpi.com/article/10.3390/medicina58010113/s1, Figure S1: Radiographic data from the 10 cases (baseline and 24 months evaluation).
