# Supplementary figures and images for: Implantoplasty Improves Clinical Parameters over a 2-Year Follow-Up: A Case Series
Source: Medicina (Kaunas). 2022 Jan 12;58(1):113. doi: 10.3390/medicina58010113 (PMC8777908; doi:10.3390/medicina58010113)

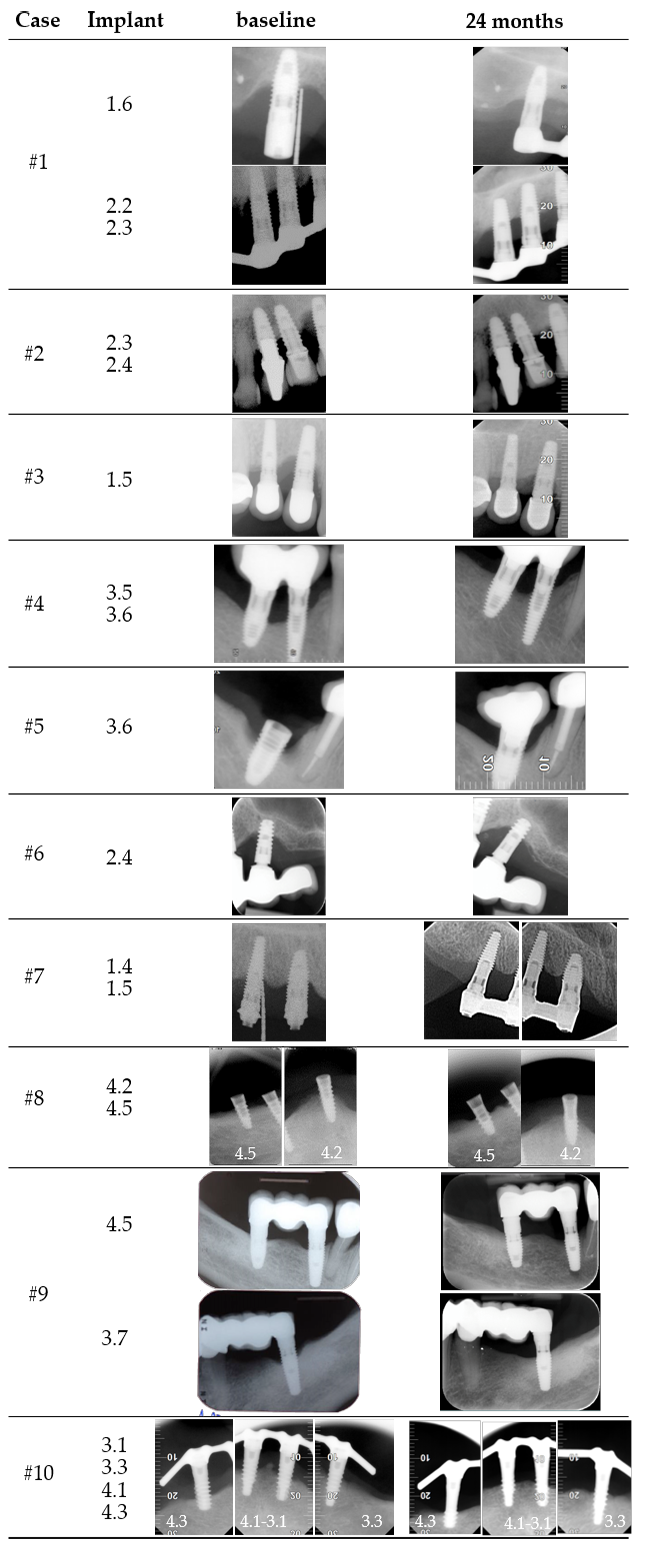

Supplement: Supplementary file 1 [file medicina-58-00113-s001.zip › medicina-1539681-supplementary.tif]
